# Impact of Reverse Empowerment and Proactive Motivations on Physicians’ Online Knowledge Sharing in Digital Platforms: Survey Study

**DOI:** 10.2196/59904

**Published:** 2024-11-29

**Authors:** Jingyuan Su, Kathy Ning Shen, Xitong Guo

**Affiliations:** 1 School of Economics and Management Zhejiang Sci-Tech University Hangzhou China; 2 College of Business and Economics Human Capital Research Center United Arab Emirates University Al Ain United Arab Emirates; 3 eHealth Research Institute School of Management Harbin Institute of Technology Harbin China

**Keywords:** physician behavior, online knowledge sharing, proactivity, patient empowerment, digital platforms, health communication

## Abstract

**Background:**

Digital platforms offer a venue for patients and physicians to exchange health information and provide health care services outside traditional organizational contexts. Previous studies have seldom focused on the factors that drive the proactivity of physicians’ online behavior. Additionally, there is limited research exploring the influence of patients on physicians’ online behavior, particularly from the perspective of patients possessing power.

**Objective:**

This study aims to investigate the effect of patient-empowering behaviors on physicians’ online knowledge sharing and uncover the potential mechanisms of proactivity. Based on the proactive motivational model and empowerment theory, we propose the existence of a reverse empowerment process, where empowerment flows from patients to physicians. We suggest that patient-empowering behaviors may drive physicians’ online knowledge sharing as a form of proactivity. Specifically, 3 proactive motivational states—knowledge-sharing self-efficacy, sharing meaning, and positive professional affect—mediate this relationship. Additionally, platform extrinsic rewards, as a contextual factor, have a moderating effect.

**Methods:**

To validate our proposed research model, we conducted a survey in China using the WJX platform, targeting physicians engaged in online knowledge sharing. The measurement instrument utilizes validated items adapted from prior research, using a 5-point Likert scale. We collected 257 valid responses, ensuring that participation was both anonymous and voluntary. Data analysis was performed in 2 stages. The first stage assessed the measurement model for reliability and validity, using the Harman 1-factor test and confirmatory factor analysis. The second stage used partial least squares-structural equation modeling to examine the direct, moderation, and mediation effects among the constructs, with bootstrapping used for significance testing. This comprehensive approach ensures a robust analysis of the proposed hypotheses and contributes to the overall validity of our research model.

**Results:**

Perceived patient-empowering behaviors significantly and positively influence physicians’ online knowledge sharing (β=0.27, *P*<.001). Knowledge-sharing self-efficacy (effect=0.06, *P*=.04), sharing meaning (effect=0.12, *P*<.001), and positive professional affect (effect=0.10, *P*=.003) each partially mediate the effect of patient-empowering behaviors on physicians’ online knowledge sharing. The overall proactive motivational states play a complete mediation role, meaning the entire indirect effect of the model is significant (effect=0.29, *P*<.001), while the direct effect in the model is nonsignificant (effect=0.07, *P*=.26). Additionally, platform extrinsic rewards significantly and negatively moderate the effect of sharing meaning on physicians’ online knowledge sharing (β=–0.13, *P*=.001).

**Conclusions:**

This study is the first to recognize and examine proactivity as an alternative mediating mechanism for physicians’ online knowledge sharing, highlighting the active role of patients in empowering physicians. It makes a significant contribution to the existing literature on empowerment, eHealth, and proactive behavior. Additionally, the findings offer valuable guidance for designing and managing digital platforms to ensure service sustainability.

## Introduction

### Background

In recent decades, there has been a significant shift, with more physicians embracing digital platforms for professional engagement. Platforms such as TikTok (ByteDance Ltd.) and HaoDF (Beijing Interactive Peak Technology Co., Ltd.) have implemented incentive mechanisms encouraging certified physicians to create and maintain personal channels for distributing health-related information. Research suggests that online information exchanges between physicians and patients offer numerous benefits, including enhanced patient outcomes, stronger physician-patient relationships, and reduced health disparities [1,2]. Online knowledge sharing generally refers to the exchange of health-related information between key stakeholders, such as physicians and patients, facilitated by digital technology platforms [3,4]. Digital platforms, particularly third-party online health platforms, provide opportunities for alternative interactions between patients and physicians beyond organizational boundaries, thereby challenging traditional health professional practices and expectations [5,6]. As a result, physicians’ participation in online knowledge sharing, such as responding to patient inquiries or posting health-related content, is considered an extra-role behavior. This activity is often carried out without organizational enforcement or immediate rewards and, in many ways, deviates from the professional practices traditionally defined by organizations and the health care industry [7].

Most prior research on physicians’ online knowledge sharing uses social exchange theory, framing it as a trade-off between the benefits and costs of participation [3,8-10]. This approach presupposes predefined expectations and exchange counterparts, which may not be present on open digital platforms. Other studies adopt an individualistic perspective, using motivation theory to investigate the intrinsic and extrinsic drivers of online knowledge sharing [5,6,11,12]. Although insightful, this perspective may overlook or underemphasize the role of altruism in physicians’ online knowledge sharing.

The adoption of digital platforms represents a paradigm shift from traditional health care, where physicians were regarded as the sole authority and patients as passive recipients of information [7]. On these platforms, patients actively influence physician behavior through actions such as information-seeking, commentary, advocacy, and even boycotts. While previous research has acknowledged the role of patients by examining platform features such as ratings, reviews, and gifts as motivators for knowledge sharing [8,11,13], there is a notable lack of studies directly addressing the significant influence patients exert on physicians’ online knowledge sharing.

This research aims to deepen the understanding of physicians’ online knowledge sharing by exploring the roles of proactivity and patient behavior. Proactivity involves actively seeking to make a difference in others’ lives or the surrounding environment to achieve better future outcomes [14]. It is characterized by self-initiated efforts (self-starting) to improve processes (situation-changing) and enhance effectiveness over the long term (future-focused) [15]. Previous studies have highlighted the role of proactivity in knowledge sharing. For example, Mittal et al [16] argued that prosocial behavior is inherently proactive, while Kang et al [17] identified users’ knowledge-sharing behaviors within a firm’s knowledge management system as examples of proactivity [17]. To encourage proactivity, both digital platforms and patients play pivotal roles. Rather than viewing patients as passive participants in physician-patient interactions, this study examines the active, empowering role that patients can assume.

Accordingly, the central research question we seek to address is: What influence does patient behavior have on the proactivity of physicians’ online knowledge sharing on digital platforms? The primary objective of this study is to investigate the impact of patient behavior on physicians’ proactive engagement in online knowledge sharing and to uncover the underlying mechanisms. Drawing on Parker et al’s [14] proactive motivation model and empowerment theory [18], we propose a comprehensive framework and research hypotheses, outlined as follows.

### Proactive Motivation Model

Most prior research relies on 2 primary perspectives to explain extra-role or prosocial behaviors, such as knowledge sharing. The first is social exchange theory, which posits that these behaviors stem from individuals weighing perceived benefits against costs [3,8-10]. However, the assumption that individuals act rationally and can accurately calculate benefits and costs is problematic, particularly given the uncertainties involved in engaging with digital platforms. Although insightful, this approach also dismisses the possibility of irrational considerations. Alternatively, basic psychological needs theory [19], rooted in self-determination theory, identifies 3 fundamental psychological needs: autonomy, competence, and relatedness. The satisfaction of these needs is thought to significantly influence overall motivation and behavior. Studies following this line of reasoning adopt a self-centric approach to explore the intrinsic factors (eg, sense of self-worth and enjoyment of helping others [5,6]) and extrinsic factors (eg, social and economic returns [11,12]) that drive extra-role or prosocial behaviors. Although useful, this approach may overlook or underemphasize other perspectives, such as altruism. Additionally, while it effectively identifies a broad range of motivational sources, it does not fully capture the motivational states that drive action.

Digital platforms offer physicians greater autonomy and incentives to explore new ways of engaging with and supporting patients [7,13]. However, participation on these platforms is voluntary, effortful, and uncertain in terms of the promised returns. What motivates physicians to overcome the resistance and challenges posed by strict professional and organizational expectations?

Consistent with the argument made by Mittal et al [16] that prosocial behavior is inherently proactive, physicians’ online knowledge sharing reflects their proactivity. This involves self-initiated efforts aimed at improving patients’ health outcomes and ensuring long-term effectiveness. According to Parker et al’s [14] framework, individual proactive behavior can be predicted by 3 proximal proactive motivational states, namely, “can do,” “reason to,” and “energized to” motivation. Can-do motivation pertains to individuals’ self-assessment of their ability to exhibit proactive behavior, or self-efficacy [6]. Reason-to motivation refers to individuals’ intrinsic drive to perceive a compelling reason to act proactively, often linked to a sense of purpose or meaning [20]. Energized-to motivation involves the activation of positive affect that enhances proactive tendencies [16].

Additionally, the impact of proactive motivational states on proactive behavior depends on individual differences and contextual factors. Notably, proactive personality, defined as an individual’s disposition to engage in active role orientations by initiating change and influencing the environment, is a key individual difference [14]. Furthermore, contextual factors, such as the initiative climate [21] and leadership [22], are thought to shape or amplify proactivity over time.

This study proposes these 3 proactive motivational pathways to better understand physicians’ proactive behavior in the context of online knowledge sharing. To explore the underlying mechanisms driving physicians’ proactivity on digital platforms, the study focuses on knowledge-sharing self-efficacy, sharing meaning, and positive professional affect as the 3 proactive motivational states for physicians. Compared with existing literature, Parker et al’s [14] framework provides a relevant and novel perspective for understanding physicians’ online knowledge sharing.

The digital platforms under examination include 2 distinct sets of contextual factors. One involves the incentive structures within the platforms, such as ranking systems or monetization features, which encourage physicians to contribute high-quality content—a dynamic driven by platform extrinsic rewards. The other pertains to the influence of patients, who act as significant social agents, representing a key contextual factor in this context [13,23]. Subsequent sections will explore the contextual impact of patients from an empowerment perspective.

### Reverse Empowerment From Patients to Physicians

Prior research has applied empowerment theory to explain the contextual influences on proactive behavior [17,24]. Empowerment refers to a process that enables individuals to gain power [25]. Traditionally, it has been viewed as the delegation of authority and decision-making power from higher- to lower-level members within an organization [26]. In a traditional health care setting, the exchange of health solutions between physicians and patients has predominantly involved unidirectional information flows from health care providers to recipients [27,28], following a paternalistic approach [29]. As a result, most research assumes that patients are empowered [30], and among the 2 key user groups of digital platforms—patients and physicians—patients have received the most attention [31-33]. Patients have become increasingly empowered through self-diagnosis [34], peer support [35], and the use of artificial intelligence to enhance health care quality [36,37]. By contrast, the perspectives of health care service providers, particularly physicians, have received limited attention [38,39]. There is a growing need to incorporate health care professionals’ views on the role of patient empowerment in digital platforms [40].

In the digital context, the traditional patient-physician relationship and paternalistic approach have been both challenged and reshaped [29]. As patients gain greater access to health information and become more actively involved in health decision-making, physicians face new challenges arising from emerging technologies and must explore their evolving roles as medical experts [41]. Therefore, it is essential to recognize and investigate the active role of patients to foster a more cooperative and effective patient-physician relationship [42].

In the marketing discipline, reverse empowerment—where service receivers empower providers—has received empirical support [18]. As customers have first-hand experience with products and services, their interactions with frontline staff can influence employees’ creativity in decision-making. A previous study identified 4 activities that can empower service providers: emphasizing the value of the information provided by service receivers, involving them in decision-making processes, expressing confidence in their abilities, and granting them increased autonomy during service interactions [18]. Such customer-initiated support has been shown to stimulate proactive behaviors in service providers. Reverse empowerment has been found to foster creativity [18] and career advancement [43] among service providers, playing a pivotal role in influencing service performance [44].

In the context of digital platforms, patients’ online engagement similarly influences providers’ actions [7]. For example, patients’ demand for medical advice and information can serve as a primary motivator for physician engagement, while patient behaviors—such as one-on-one chats, information seeking, advocacy, and feedback—significantly impact physicians’ actions. Therefore, we consider patient behaviors a critical contextual factor in examining their influence on physicians’ online knowledge sharing. Similar to customer-empowering behaviors, patient-empowering behaviors are defined as actions taken by patients that inspire physicians and enable them to take steps toward achieving desired outcomes on digital platforms.

### Research Model and Hypotheses

#### The Impact of Perceived Patient-Empowering Behaviors on Physicians’ Online Knowledge Sharing

**Figure 1:**
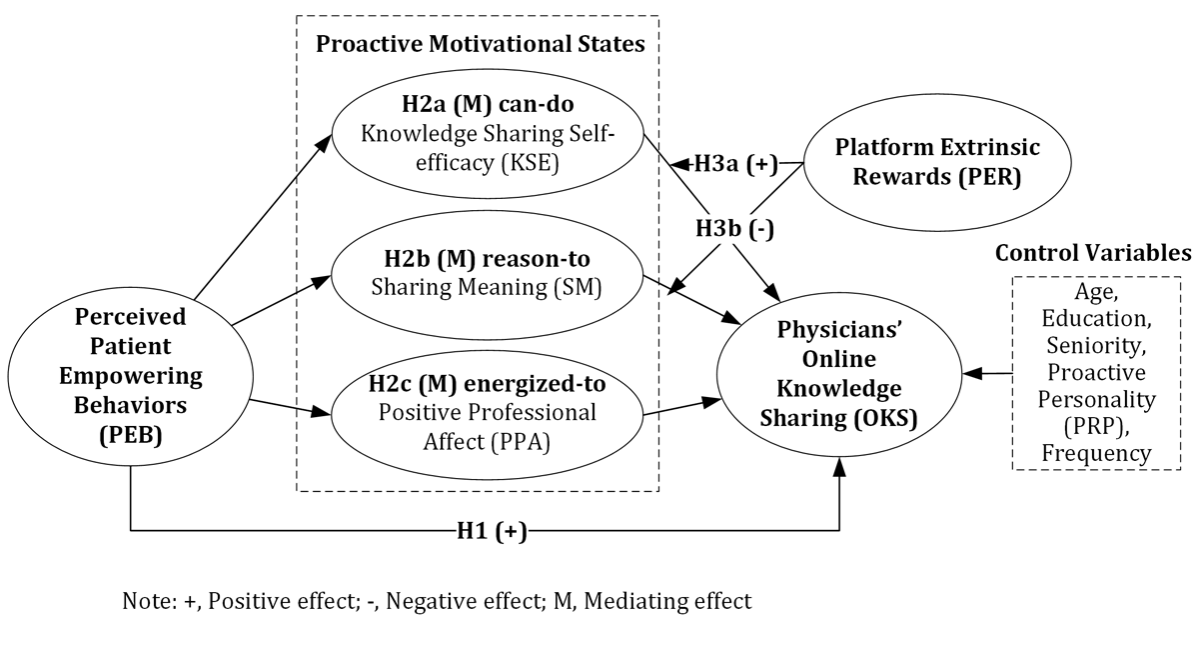
Research model and hypotheses.

Figure 1 illustrates the proposed research model based on the above literature discussion. Empowering behaviors, in general, serve as positive responses that motivate the replication of specific actions [22]. Therefore, when physicians’ online knowledge-sharing behaviors are reinforced by positive feedback from patients, they are more likely to sustain these behaviors, leading to a cycle of continuous and beneficial online knowledge-sharing practices.

Drawing from reverse empowerment research in marketing [18], we identify 4 distinct online actions enacted by patients as empowering behaviors: highlighting the usefulness of physicians’ information, involving physicians in the decision-making process, expressing confidence in physicians’ abilities, and granting physicians autonomy in knowledge sharing. First, usefulness refers to the perceived value that patients attribute to online health information in addressing their health-related issues and queries. Prior research has highlighted the positive impact of perceived usefulness on knowledge-sharing behavior within virtual communities [45]. Second, patients increasingly view digital platforms as valuable tools for decision-making, largely due to the provision of unbiased information [46]. Effective assistance from physicians in decision-making requires patients to share their concerns and health status. Third, patients are more likely to share their health information with physicians they trust and perceive as competent [47]. The perception of being trusted enhances a physician’s confidence and interest in online knowledge sharing, thereby fostering sustained engagement from physicians [48,49]. Lastly, online knowledge sharing is not limited to physicians’ domain expertise as solution providers. As Mesko et al [41] suggested, “the characteristics of a physician-as-idol could shift from self-confident to curious, from rule follower to creative, and from lone hero to team worker.” Encouragement from patients can stimulate physicians to explore and become more creative. Therefore, given the pivotal role of patients as key social entities on digital platforms, their empowering behaviors are likely to stimulate physicians’ participation in online knowledge sharing. We propose the following:

- Hypothesis 1: Perceived patient-empowering behaviors are positively associated with p
  hysicians’ online knowledge sharing.

#### Mediating Role of Proactive Motivational States

Digital platforms can flatten organizational hierarchies and transcend geographic boundaries, enabling physicians of varying ranks and locations to engage more actively. While patient-empowering behaviors enhance the likelihood of physicians’ participation in online knowledge sharing, the unique nature of interactions on these platforms requires the identification of contingencies that may influence this relationship. Unlike traditional face-to-face interactions, digital platform exchanges are not constrained by treatment relationships, and there is often an abundance of information available about both parties. The perception of physicians as valuable or helpful is shaped not only by their qualifications and professional experience but also by their online content and communication with patients [8,13]. Therefore, for patient-empowering behaviors to drive proactive knowledge sharing, they must instigate internal transformations in physicians.

Anchored in the proactive motivation model [14], this study defines 3 proactive motivational states that drive physicians’ online knowledge sharing: can-do motivation (knowledge-sharing self-efficacy), reason-to motivation (sharing meaning), and energized-to motivation (positive professional affect). These motivational states are nurtured by patient-empowering behaviors, which in turn influence physicians’ actions on digital platforms. The framework highlights not only how physicians feel empowered but also how they transform this empowerment into proactive behaviors.

First, knowledge-sharing self-efficacy, as a form of can-do motivation, refers to physicians’ confidence in their professional abilities and their effectiveness in communicating online to share valuable knowledge with patients [14]. Patient-empowering behaviors, such as demonstrating patients’ confidence in physicians’ capabilities during service delivery, enable physicians to perceive themselves as possessing high levels of expertise, skill, and competence. This positive feedback from patients contributes to an increase in physicians’ self-efficacy in knowledge sharing. Research shows that individuals with higher levels of knowledge-sharing self-efficacy are more likely to offer valuable advice and support to others [50]. This is because a strong sense of efficacy fosters a heightened sense of responsibility to use their expertise for the benefit of others, as demonstrated in studies on self-efficacy and prosocial behavior [51]. Therefore, we hypothesize:

- Hypothesis 2a: Knowledge-sharing self-efficacy, as can-do motivation, mediates the relationship between perceived patient-empowering behaviors and physicians’ online knowledge sharing.

Second, sharing meaning, as a form of reason-to motivation, reflects the significance and value that physicians attribute to their online behavior [14]. Physicians who perceive greater meaning in sharing knowledge are likely to experience increased self-worth and fulfillment, which can enhance the quality of their service provision [5,9]. Individuals who find meaning in their actions are more inclined to engage in behaviors that reinforce this positive self-perception [52], such as sharing knowledge with others. Moreover, when patient-empowering behaviors highlight the value and effectiveness of physicians’ contributions, it reinforces the importance of their role, deepening their commitment to knowledge sharing. This heightened sense of significance not only makes physicians feel that their contributions are meaningful but also fosters a sense of responsibility and enthusiasm to continue their online knowledge sharing activities. Hence, we hypothesize the following:

- Hypothesis 2b: Sharing meaning, as reason-to motivation, mediates the relationship between perceived patient-empowering behaviors and physicians’ online knowledge sharing.

Lastly, as energized-to motivation, positive professional affect refers to the positive emotions and attitudes physicians associate with their professional experiences [53]. As participation in digital platforms is voluntary and can sometimes be perceived as a distraction from core responsibilities, physicians need to experience positive emotions related to their professional role to remain engaged. Positive professional affect also represents the internalization of online knowledge sharing into physicians’ professional identities, helping reconcile the tension between online presence and traditional professional expectations [54]. This factor can influence physicians’ proactivity by shaping their professional aspirations, aligning their actions with their core professional values and goals [21]. When physicians experience a strong sense of positive affect toward their profession, they are more likely to engage in proactive behaviors. These activities reinforce their positive self-concept and contribute to their professional fulfillment, thereby energizing their motivation to engage in online knowledge sharing. Hence, we hypothesize the following:

- Hypothesis 2c: Positive professional affect, as energized-to motivation, mediates the relationship between perceived patient-empowering behaviors and physicians’ online knowledge sharing.

#### Moderating Role of Extrinsic Rewards

Platform extrinsic rewards serve to validate individuals’ capabilities and reinforce their expectations regarding online knowledge sharing [5,9,11,23]. Physicians with high self-efficacy are confident in their ability to share knowledge online. Consequently, in the presence of favorable platform extrinsic rewards, they are more likely to be motivated to engage in online knowledge sharing and confident in the outcomes of their efforts, thus establishing a cycle of positive reinforcement through a self-fulfilling prophecy [55]. Previous research has indicated that rewards effectively moderate the impact of self-efficacy on both effort [56] and performance [57]. Hence, we hypothesize the following:

- Hypothesis 3a: Platform extrinsic rewards positively moderate the relationship between knowledge-sharing self-efficacy and physicians’ online knowledge sharing.

According to the motivation crowding theory, platform-based extrinsic incentives have the potential to displace an individual’s intrinsic motivation to act based on their own internal needs [54]. Similarly, cognitive evaluation theory posits that when external rewards are perceived as controlling, they can undermine intrinsic motivation by shifting the perceived locus of causality from internal to external. In the context of physicians’ online knowledge sharing, platform extrinsic rewards can diminish contributors’ intrinsic drive, thereby impeding their participation. This aligns with studies suggesting that external interventions can negatively moderate the connection between intrinsic benefits and the intention to contribute to online feedback systems [58]. As sharing meaning reflects a sense of self-worth, it represents an intrinsic factor influencing physicians’ behaviors [14]. Hence, we hypothesize the following:

- Hypothesis 3b: Platform extrinsic rewards negatively moderate the relationship between sharing meaning and physicians’ online knowledge sharing.

Lastly, the impact of platform extrinsic rewards on moderating the relationship between positive professional affect and online knowledge sharing may yield ambivalent outcomes. On the one hand, platform extrinsic rewards tied to the quality and quantity of physicians’ online knowledge sharing could empower physicians with high levels of positive affect in their occupations, motivating them to increase their contributions and achieve their desired occupational expectations and goals [21]. On the other hand, similar to the substitution effect of extrinsic motivation on intrinsic motivation [59], the pursuit of extrinsic rewards could potentially diminish the intrinsic drive derived from the positive affect inherent to physicians’ occupation. The moderating effect of platform extrinsic rewards is likely contingent on the magnitude of the rewards. However, due to limited knowledge of the actual extrinsic rewards experienced by physicians on digital platforms and the ambiguous evidence regarding their moderating effect, we refrain from formulating a hypothesis.

### Study Objectives

The main objective of this research is to explore the influence of patient-empowering behaviors on physicians’ online knowledge sharing, examine the mediating role of proactive motivational states, namely, can-do, reason-to, and energized-to motivation in this relationship, and assess the moderating effect of platform extrinsic rewards on the connection between proactive motivational states and physicians’ online knowledge sharing. To empirically test our theoretical framework, we collected data via a survey targeting physicians engaged in online knowledge sharing and analyzed the data using structural equation modeling. The results provide strong support for the proposed research model.

## Methods

### Ethical Considerations

The study was conducted in accordance with the Institutional Review Board of the School of Management at Harbin Institute of Technology. All participants were thoroughly informed about the nature of the study and the voluntary nature of their participation in the web-based survey. They were assured that all data collected would be treated with strict confidentiality and managed in accordance with established ethical standards. The survey was anonymous, with no personal or identifying information being collected or accessible to the researchers.

### Data Collection

This survey was conducted in China. Three companies, with physicians as their clients, were approached and agreed to facilitate data collection. In these companies, salespersons maintain direct service and business relationships with physicians. Approximately 50 medical salespersons were randomly selected from these companies to support the data collection.

The questionnaire was designed in an electronic format using the WJX platform. Salespersons distributed the questionnaire to physicians via WeChat (Tencent Holdings Limited) or provided it during offline visits. Physicians could access the questionnaire by scanning a QR code or clicking on a forwarded link through WeChat on their mobile phones. Additionally, a snowball sampling method was used, where participating salespersons and physicians were asked to forward the questionnaire to other physicians they knew.

We included a screening question at the beginning of the questionnaire to identify physicians with no experience in online knowledge sharing. Physicians without this experience were excluded from the sample. To ensure anonymity, no personal or identifying information was collected from participants. Additionally, no incentive mechanism was offered to the salespersons or respondents. To detect potential errors associated with the online survey, we included reverse-coded questions and ensured that participation was entirely voluntary and anonymous. To prevent duplication, we collected a WeChat ID or phone number as an identifier, ensuring that each physician could complete the questionnaire only once.

A total of 571 responses were collected over 1 month, including 327 physicians with experience in online knowledge sharing and 244 without such experience, as indicated by the screening question. Invalid responses—such as those with no response variance, incomplete answers, or contradictory information—were examined and filtered out, leaving 257 valid responses for data analysis and hypothesis testing. More detailed information on data collection issues can be found in Multimedia Appendix 1 [60].

### Measurement Instrument

To validate the research model, a survey study was conducted using items adapted from previous research to ensure both validity and contextual relevance. A 5-point Likert scale was used, with responses ranging from 1=strongly disagree to 5=strongly agree. Physicians’ online knowledge sharing was measured with 4 items adapted from Zhang et al [5]. The scale for patient-empowering behaviors was derived from Dong et al’s [18] customer-empowering behaviors scale, retaining key aspects of their original framework. Four dimensions, each with 2 items, were used to measure perceived patient-empowering behaviors, except for the dimension of autonomy. One autonomy item (“Customer allowed me to make important decisions to satisfy his/her needs”) was excluded from the research context, as physicians do not make decisions for patients but instead provide advice. In total, 7 items were used to assess perceived patient-empowering behaviors. The 3 dimensions of proactive motivational states were measured using 3 items for knowledge-sharing self-efficacy, based on Lin et al [50]; 3 items for sharing meaning, adapted from Spreitzer [61]; and a measurement for positive professional affect, adapted from Watson et al [62]. Platform extrinsic rewards were assessed with 3 items from Yang and Lai [63].

To examine how physicians’ characteristics may influence their online behaviors, control variables were considered. Proactivity personality was measured using an adaptation of the scale from Parker et al [14]. Nominal scales were used to assess seniority, age, frequency, and education level.

To ensure the validity of the measurement during the translation of the instrument from English to Chinese, we used a back-translation method. Three experts in the field of information systems reviewed the instrument for clarity, question wording, ease of understanding, logical consistency, item sequence, and contextual relevance, suggesting minor modifications to the wording and item order. A pilot study involving 40 physicians was then conducted using the revised questionnaire, which enhanced clarity and reduced ambiguities. We recorded the time taken by physicians to complete the survey as a criterion for evaluating response quality. Items with unsatisfactory loadings were removed as a result. Detailed information about the survey and its items is provided in Multimedia Appendix 2.

### Statistical Analysis Methods

This study utilizes SPSS (version 26; IBM Corp.) and SmartPLS (version 3.0; SmartPLS GmbH) software to conduct multiple stages of data analysis.

In the first stage, we conducted a measurement model examination to assess the validity and reliability of the constructs in our study. First, we checked for potential selection bias by comparing early and late respondents across all variables. Second, to examine common method bias, we performed the Harman 1-factor test using SPSS [64]. Third, confirmatory factor analysis was conducted to assess the reliability and validity of the research model. All factor loadings exceeded the 0.70 threshold, indicating good convergent validity. Composite reliability and Cronbach α values were above 0.70, and the average variance extracted values surpassed 0.50, demonstrating strong internal consistency and reliability. Additionally, the square root of each average variance extracted exceeded the correlations with other variables, confirming discriminant validity. Finally, collinearity among predictors was assessed, with variance inflation factor values below the 3.3 threshold, indicating no multicollinearity issues [65].

In the second stage, partial least squares-structural equation modeling was used to examine the structural model, including direct, moderation, and mediation effects, in order to test the proposed hypotheses [66]. Partial least squares-structural equation modeling, a robust technique based on bootstrapping, was selected for its flexibility in handling less stringent distributional assumptions and smaller sample sizes compared with other methods. The structural model was assessed through path estimations and the model’s explanatory power. A bootstrapping resampling approach with 5000 resamples was used to evaluate the significance of the paths, providing the explained variance of endogenous variables (*R*^2^), standardized path coefficients (β), and the significance of these estimates (*P* value). Additionally, to rigorously test the mediation effects of proactive motivational states, we used bootstrapping, which does not require the assumption of normality for the sampling distribution. Finally, interaction terms were used to statistically test the moderating effects. The moderating role was visually depicted through a multigroup analysis. The data set was divided into 2 groups based on the mean value of platform extrinsic rewards, creating high- and low-reward subsets. The difference in slopes between these 2 groups demonstrated how platform extrinsic rewards moderated the relationship.

## Results

### Descriptive Analysis

Table 1 presents the demographic characteristics of the respondents. The sample (N=257) showed a gender balance, with 103 male physicians (40.1%) and 154 female physicians (59.9%). The majority of respondents were aged between 31 and 40 years (132/257, 51.4%). Most platforms provided payment consultation services, ranking systems, or gift incentives to encourage physicians’ online engagement. The frequency with which respondents used these platforms over the past 3 months varied among physicians.

**Table 1:** Demographic characteristics of sample participants (N=257).

Demographic variables			Values, n (%)
**Gender**			
	Male	103 (40.1)	
	Female	154 (59.9)	
**Age (years)**			
	<30	37 (14.4)	
	31-40	132 (51.4)	
	41-50	65 (25.3)	
	51-60	20 (7.8)	
	>60	3 (1.2)	
**Education level**			
	Bachelor’s degree	109 (42.4)	
	Master’s degree	98 (38.1)	
	PhD degree	50 (19.5)	
**Seniority**			
	Resident physician	45 (17.5)	
	Attending physician	105 (40.9)	
	Deputy chief physician	76 (29.6)	
	Chief physician	31 (12.1)	
**Frequency of** **online** **knowledge sharing in the last 3 months**			
	Once a day	25 (9.7)	
	Once every 3 days	30 (11.7)	
	Once a week	50 (19.5)	
	Once every 2 weeks	23 (8.9)	
	Once a month	129 (50.2)	
**Digital** **platforms** **(multichoice)**			
	HaoDF	80 (31.1)	
	Dingxiang Doctor	117 (45.5)	
	Weiyi	34 (13.2)	
	Chunyu Doctor	37 (14.4)	
	PingAn Doctor	43 (16.7)	
	Others	90 (35.0)	

### Measurement Model

First, the results of the selection bias test indicate no significant differences between early and late respondents (online knowledge sharing, dependent variable, *P*=.92). Second, the results of the Harman 1-factor test revealed that the first and largest factor of the unrotated solution explained 41.3% of the total variance, which is below the 50% threshold, suggesting no evidence of common method bias in this study. Third, as shown in Tables 2 and 3, the results demonstrated good convergent validity, strong internal consistency, reliability, and discriminant validity. The variance inflation factor values ranged from 1.064 to 2.167, all below the threshold of 3.3, indicating no multicollinearity issues in this study.

**Table 2:** Construct reliability and convergent validity.

Constructs		Factor loadings	Composite reliability	Average variance extracted	Cronbach α
**OKS^a^**					
	OKS1	0.900	0.936	0.786	0.909
	OKS2	0.864	N/A^b^	N/A	N/A
	OKS3	0.892	N/A	N/A	N/A
	OKS4	0.889	N/A	N/A	N/A
**PEB^c^**					
	PEB1	0.786	0.920	0.620	0.898
	PEB2	0.810	N/A	N/A	N/A
	PEB3	0.773	N/A	N/A	N/A
	PEB4	0.766	N/A	N/A	N/A
	PEB5	0.793	N/A	N/A	N/A
	PEB6	0.795	N/A	N/A	N/A
	PEB7	0.790	N/A	N/A	N/A
**KSE^d^**					
	KSE1	0.812	0871	0.693	0.780
	KSE2	0.867	N/A	N/A	N/A
	KSE3	0.817	N/A	N/A	N/A
**SM^e^**					
	SM1	0.890	0.921	0.795	0.871
	SM2	0.886	N/A	N/A	N/A
	SM3	0.899	N/A	N/A	N/A
**PPA^f^**					
	PPA1	0.898	0.973	0.798	0.968
	PPA2	0.871	N/A	N/A	N/A
	PPA3	0.916	N/A	N/A	N/A
	PPA4	0.884	N/A	N/A	N/A
	PPA5	0.892	N/A	N/A	N/A
	PPA6	0.918	N/A	N/A	N/A
	PPA7	0.924	N/A	N/A	N/A
	PPA8	0.898	N/A	N/A	N/A
	PPA10	0.838	N/A	N/A	N/A
**PER^g^**					
	PER1	0.879	0.911	0.773	0.853
	PER2	0.880	N/A	N/A	N/A
	PER3	0.879	N/A	N/A	N/A
**PRP^h^**					
	PRP1	0.916	0.890	0.731	0.839
	PRP2	0.906	N/A	N/A	N/A
	PRP4	0.733	N/A	N/A	N/A

^a^OKS: online knowledge sharing.

^b^N/A: not applicable.

^c^PEB: patient-empowering behaviors.

^d^KSE: knowledge sharing self-efficacy.

^e^SM: sharing meaning.

^f^PPA: positive professional affect.

^g^PER: platform extrinsic rewards.

^h^PRP: proactive personality.

**Table 3:** Discriminant validity^a^.

Constructs	OKS^b^	PEB^c^	SM^d^	KSE^e^	PPA^f^	PER^g^	PRP^h^
OKS	0.887	—^i^	—	—	—	—	—
PEB	0.471^j^	0.788	—	—	—	—	—
SM	0.635^j^	0.427^j^	0.892	—	—	—	—
KSE	0.473^j^	0.533^j^	0.392^j^	0.833	—	—	—
PPA	0.554^j^	0.526^j^	0.430^j^	0.474^j^	0.893	—	—
PER	0.635^j^	0.415^j^	0.505^j^	0.379^j^	0.540^j^	0.879	—
PRP	0.086	0.110	0.07	0.074	–0.045	–0.036	0.855

^a^Diagonal numbers are average variance extracted square root, others are correlation coefficients.

^b^OKS: online knowledge sharing.

^c^PEB: patient-empowering behaviors.

^d^SM: sharing meaning.

^e^KSE: knowledge sharing self-efficacy.

^f^PPA: positive professional affect.

^g^PER: platform extrinsic rewards.

^h^PRP: proactive personality.

^i^Not applicable.

^j^*P*<.01.

### Structural Model

**Figure 2:**
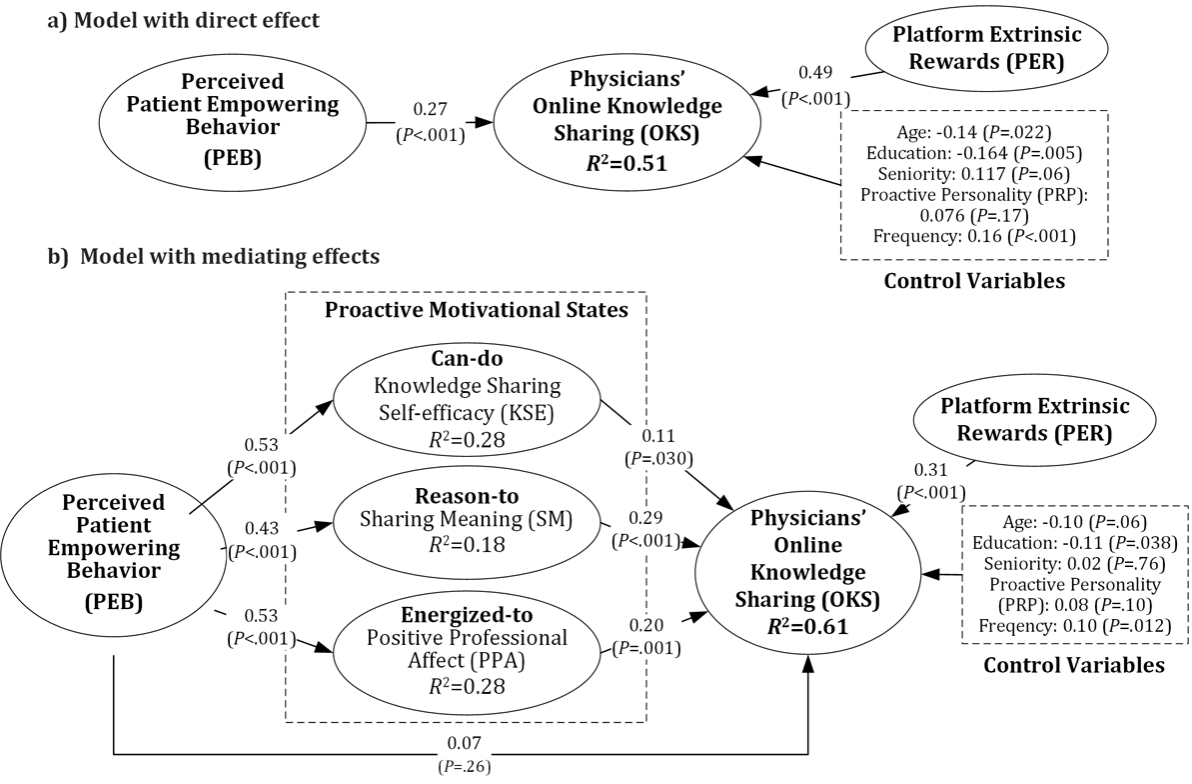
Analysis results of the structural model. (A) Model with direct effects; (B) model with mediating effects.

As illustrated in model A of Figure 2, the direct effect demonstrates that perceived patient-empowering behaviors are positively associated with online knowledge sharing (β=0.27, *P*<.001). Model A explains 51% of the variance in online knowledge sharing, with the direct effect contributing an additional 6.2% variance compared with the baseline model, which includes only the control variables. Therefore, hypothesis 1 is supported.

Regarding the control variables, physicians’ age and education level are negatively correlated with online knowledge sharing, whereas the frequency of participation and platform extrinsic rewards show positive correlations with online knowledge sharing.

### Mediation Effect

Model B, as delineated in Figure 2, demonstrates a positive correlation between perceived patient-empowering behaviors and the 3 proactive motivational states: knowledge-sharing self-efficacy (β=0.53, *P*<.001), sharing meaning (β=0.43, *P*<.001), and positive professional affect (β=0.53, *P*<.001). Furthermore, these 3 proactive motivational states—knowledge-sharing self-efficacy, sharing meaning, and positive professional affect—are positively associated with online knowledge sharing among physicians, with standardized path coefficients of 0.11 (*P*=.03), 0.29 (*P*<.001), and 0.20 (*P*=.001), respectively. The explanatory power of this model accounts for 61% of the variance in online knowledge sharing, representing a 16% increase in variance explained over the baseline model that included only control variables.

The application of this nonparametric resampling method reveals significant findings for the entire indirect effect (effect=0.29, *P*<.001), while the direct effect is statistically insignificant (effect=0.07, *P*=.26). These results underscore the complete mediation role of proactive motivational states in the relationship between physicians’ perceived patient-empowering behaviors and their engagement in online knowledge sharing. Additionally, the partial mediation effects are significant for knowledge-sharing self-efficacy (effect=0.06, *P*=.04), sharing meaning (effect=0.12, *P*<.001), and positive professional affect (effect=0.10, *P*=.003). These findings indicate that each of these motivational states individually mediates the relationship between physicians’ perceived patient-empowering behaviors and their online knowledge sharing to some extent, thereby supporting hypothesis 2a-c (Table 4).

**Table 4:** Results of testing mediation effects for proactive motivational states.

Mediation effect			Effect		*P* value		Bias-corrected 95% CI
Total effect			0.36		<.001		0.23 to 0.48
Total indirect effect			0.29		<.001		0.19 to 0.39
**Specific indirect effect**							
	Knowledge sharing self-efficacy	0.06		.04		0.01 to 0.11	
	Sharing meaning	0.12		<.001		0.07 to 0.19	
	Positive professional affect	0.10		.003		0.04 to 0.16	
Direct effect			0.07		.26		–0.05 to 0.20

### Moderating Effect

The analysis presented in Table 5 evaluates the moderating effect of platform extrinsic rewards. A single significant finding emerges, specifically regarding the interaction between sharing meaning and online knowledge sharing (β=–0.13, *P*=.001). This result supports hypothesis 3b, while no evidence was found to support hypothesis 3a.

**Table 5:** The moderating effect of platform extrinsic rewards.

Path	Coefficient (β)	*P* value
PER^a^ × KSE^b^→OKS^c^	–0.03	.57
PER × SM^d^→OKS	–0.13	.001

^a^PER: platform extrinsic rewards.

^b^KSE: knowledge sharing self-efficacy.

^c^OKS: online knowledge sharing.

^d^SM: sharing meaning.

**Figure 3:**
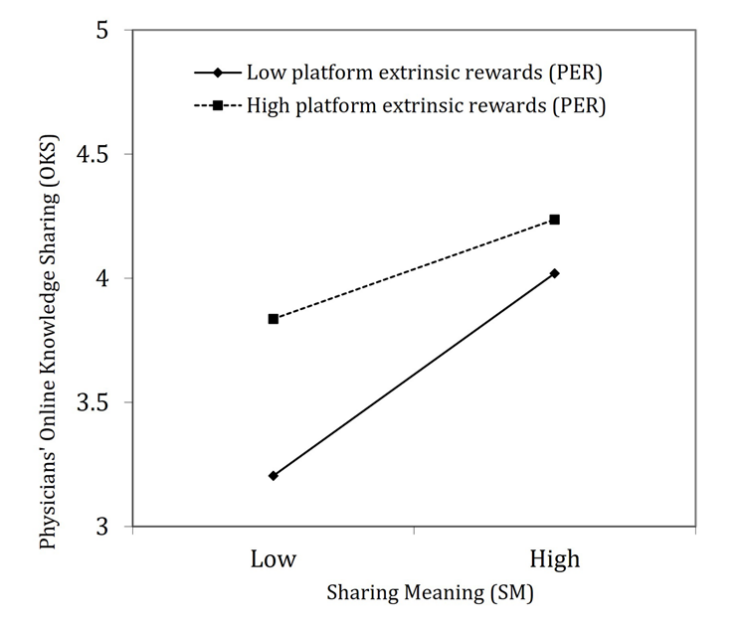
Interaction between platform extrinsic rewards and sharing meaning in influencing physicians’ online knowledge sharing.

Figure 3 graphically illustrates the moderating role of platform extrinsic rewards in the relationship. The figure highlights a nuanced dynamic in how the level of platform extrinsic rewards affects physicians’ online knowledge sharing. Specifically, with a higher level of platform extrinsic rewards (depicted by the dotted line in Figure 3), increases in sharing meaning correspond to a more gradual change in physicians’ online knowledge sharing. Conversely, in scenarios with lower platform extrinsic rewards (depicted by a solid line in Figure 3), increases in sharing meaning result in a more pronounced rise in physicians’ online knowledge sharing.

## Discussion

### Principal Findings

This study highlights how patient behaviors can enhance and empower physicians’ online knowledge sharing, mediated by proactive motivational states, including knowledge-sharing self-efficacy (can-do motivation), sharing meaning (reason-to motivation), and positive professional affect (energized-to motivation). Furthermore, platform extrinsic rewards, as a contextual factor, negatively moderate the relationship between sharing meaning and online knowledge sharing.

Specifically, first, our empirical evidence supports the phenomenon of reverse empowerment from patients to physicians within digital platforms. Encounters with patient-empowering behaviors across 4 dimensions make physicians more inclined to engage in online knowledge sharing. This relationship aligns with findings by Dong et al [18] and Zhang et al [13], although, in this context, the behavior of the “service provider” (knowledge contributors) is oriented toward a future exchange.

Second, our results further validate the mediating role of proactive motivational states, triggered by perceived patient-empowering behaviors. Notably, these proactive motivational states fully mediate the influence of perceived patient-empowering behaviors on online knowledge sharing, especially when considering the potential effects of platform extrinsic rewards. Each motivational state—knowledge-sharing self-efficacy, sharing meaning, and positive professional affect—partially contributes to the overall mediating effect. These results confirm the viability of conceptualizing physicians’ online knowledge sharing as an aspect of proactive behavior, presenting proactivity as an alternative framework to explain physicians’ engagement in online knowledge sharing [17]. The considerable mediation effect exerted by proactive motivational states underscores the desired nature of social dynamics between patients and physicians on digital platforms. It highlights the impactful role of patient-driven empowering actions in enhancing physicians’ perceived sharing meaning, knowledge-sharing self-efficacy, and positive professional affect, thereby amplifying knowledge dissemination.

Third, our findings align with the negative moderating effect of platform extrinsic rewards on the link between sharing meaning and online knowledge sharing, consistent with existing literature suggesting that extrinsic motivators may undermine intrinsic motivation [59]. The absence of a moderating effect of platform extrinsic rewards on the impact of knowledge-sharing self-efficacy could be attributed to the predominantly high self-efficacy levels among the physicians surveyed. This suggests a possible limit to the effectiveness of platform extrinsic rewards, as individuals with already high self-efficacy do not seem to experience further enhancement in their online knowledge-sharing behaviors.

Regarding control variables, the significant positive correlation between platform extrinsic rewards and online knowledge sharing corroborates previous studies within the knowledge-sharing motivation theory domain [5,9]. The demographic characteristics of participating physicians indicate that digital platforms tend to attract younger and less experienced physicians. The pursuit of professional advancement, the acquisition of broader experience, and the availability of such platforms might serve as key drivers for platform engagement.

### Theoretical Implications

Our findings offer 3 important contributions to the existing literature. First, our study enriches the existing empowerment literature by investigating the reverse empowerment process from patients to physicians. Traditional scholarship on empowerment predominantly conceptualizes the empowerment process as emanating from the more authoritative party toward those in subordinate positions, such as empowerment initiatives from organizations [25,61], leaders [67], or service providers [68] directed at employees, subordinates, or service recipients. The digital environment, however, disrupts traditional information hierarchies, endowing previously perceived “lesser-powered” entities with empowerment through the proactive seeking and dissemination of information. While marketing research has started to probe into consumers acting as a prominent source of empowerment to motivate service employees toward taking control of the service process [18], the notion of patients empowering physicians has not garnered sufficient attention. Our investigation stands as a new effort to scrutinize and validate the substantial influence patients wield in elevating physicians on digital platforms, thereby completing the loop of empowerment and augmenting comprehension of the genesis of an empowerment cycle and productive patient-physician dynamics.

Second, this study enhances the eHealth literature by characterizing physicians’ online knowledge sharing as proactive conduct. The advent of digital technology presents challenges and opportunities to traditional professional roles and workplace settings. Proactive behavior, epitomized as self-initiated, future-oriented actions aimed at altering one’s work environment, roles, or self, is vital for individuals and organizations to navigate the demands of a rapidly evolving milieu [69]. Despite extensive coverage by management researchers aiming to broaden the scope of job performance within the confines of organizational settings [70], the proactive aspect of physicians’ online knowledge sharing has largely been overlooked. By applying the proactive behavior paradigm to eHealth research within the ambit of open digital platforms, our study not only deepens the existing understanding but also crucially opens new avenues for future exploration.

Moreover, our examination advances the understanding of proactive behavior within a novel milieu and its contextual determinants. Previous inquiries have scrutinized the contextual variables influencing proactive behaviors within formal organizational structures, such as leadership, work design, and social dynamics [14], which may manifest differently within the context of digital health platforms. Given the reciprocal nature of the physician-patient relationship, social stimuli (perceived patient-empowering behaviors) alongside platform design attributes (platform extrinsic rewards) emerge as catalysts for physicians to adopt proactive stances within digital platforms. By identifying and empirically demonstrating the significance of situational conditions that foster proactive behaviors among physicians in this emergent context, our analysis makes a valuable contribution to the discourse on proactive conduct.

### Practical Implications

Considering the increasing popularity and adoption of digital channels by traditional health care service providers, the findings of this study offer valuable insights into constructing and sustaining effective digital platforms. First, it is crucial for platforms to view patients or general participants not merely as information seekers but as key catalysts in the empowerment cycle. It is essential, therefore, to acknowledge and incentivize their empowering actions, which play a crucial role in sustaining the ongoing engagement of physicians and other experts. For instance, to increase patient-empowering behaviors, managers might enhance patients’ ability to affirm the competence of physicians or provide simpler ways for patients to acknowledge and commend other physicians’ contributions. Furthermore, platforms should focus on improving the visibility of relevant patient-empowering behaviors to physicians, such as by highlighting the positive outcomes of online consultations.

Second, while the deployment of platform extrinsic rewards is critical, it alone may prove inadequate in catalyzing physicians’ proactive engagement. It is advisable for platforms to incorporate proactive emotional states into their metrics for designing, assessing, and refining the efficacy of incentives. Additionally, given that patient-empowering behaviors attenuate the mediating influence of sharing meaning, it becomes necessary for platforms to strike a harmonious balance between patient-empowering behaviors and strategies that promote them.

Lastly, the phenomenon of reverse empowerment may have implications for clinical practices, especially in contexts where physicians engage in regular interactions with patients. There is an opportunity for patients to collaborate with physicians during the provision of clinical services. Consequently, clinical institutions should strive to raise patients’ awareness of how their empowering behavior could lead to more proactive services from physicians, thereby fostering a more dynamic and reciprocal patient-physician interaction.

### Limitations and Future Directions

Although this research provides valuable insights into both theoretical and practical domains, several limitations underscore the need for further scholarly exploration. First, the scope of this study is confined to third-party open platforms, excluding a comprehensive analysis of the various organizational affiliations of physicians. In light of the growing embrace and valuation of digital channels by health care providers, the findings of this study may have broader implications for physicians’ professional performance. Future research should investigate organizational platforms and explore potential interactions between online participation and organizational factors in driving physicians’ proactive efforts.

Second, to attract domain expertise and encourage continuous engagement, platforms often compete by designing various incentives. While this study considers extrinsic rewards as perceived by physicians to account for varying exposure levels to different platform incentives, it would be beneficial to expand research to directly examine these platform incentives and their impact on patient-physician interactions.

Third, given the cross-sectional design of our study, the ability to empirically substantiate causality is limited. Nonetheless, our findings support the value of considering proactivity as an alternative mechanism and underscore the significant effect of perceived patient-empowering behaviors. This design allowed us to capture physicians’ personal perceptions and focus on the internal mechanisms driving their behavior. While our study sets the stage for understanding these perceptual dynamics, it also paves the way for future research to track dynamic behavioral changes using a longitudinal design, further clarifying the time-based causal effects between physicians’ proactive behavior changes and patient behavior.

### Conclusions

Drawing upon proactive motivation theory and the concept of the reverse empowerment process, this study conceptualizes physicians’ online knowledge sharing as a form of proactive behavior and investigates the underlying mechanisms by identifying contextual factors and empirically validating their impact on physicians’ online knowledge sharing. The findings suggest that the reverse empowerment process, specifically perceived patient-empowering behaviors, can facilitate physicians’ online knowledge sharing by fostering proactive motivational states such as knowledge-sharing self-efficacy, sharing meaning, and positive professional affect. These motivational states emerge as pivotal mediators, while platform extrinsic rewards may attenuate the positive relationship between sharing meaning and online knowledge sharing. This study makes significant contributions to the existing literature on empowerment, eHealth, and proactive behavior. The findings also offer valuable insights for developing strategies to ensure the sustainability of services in the design and management of digital health platforms.

## Appendix

Multimedia Appendix 1 The Checklist for Reporting Results of Internet E-Surveys (CHERRIES).

Multimedia Appendix 2 Measurement scales.
