# Supplementary material for: Impact of Reverse Empowerment and Proactive Motivations on Physicians’ Online Knowledge Sharing in Digital Platforms: Survey Study
Source: J Med Internet Res. 2024 Nov 29;26:e59904. doi: 10.2196/59904 (PMC11661403; doi:10.2196/59904)
Supplement: Multimedia Appendix 1 [file jmir_v26i1e59904_app1.docx]

**Multimedia Appendix 1**

Table. Checklist for Reporting Results of Internet E-Surveys (CHERRIES)

| Checklist for Reporting Results of Internet E-Surveys (CHERRIES) | | |  |
| --- | --- | --- | --- |
| Item Category | Checklist Item | Explanation | JMIR ms#59904 Paper |
| Design |  |  |  |
|  | Describe survey design | Describe target population, sample frame. Is the sample a convenient sample? (In “open” surveys this is most likely.) | The target population of the study is physicians engaged in online knowledge sharing on digital platforms. The study collected responses from a sample of 257 physicians who were active on these platforms and had experience with online knowledge sharing. An online anonymous questionnaire was used to collect data from these participants. The sample appears to be a convenience sample, as participation was voluntary, and respondents self-selected into the study. |
| IRB (Institutional Review Board) approval and informed consent process |  |  |  |
|  | IRB approval | Mention whether the study has been approved by an IRB. | The study was conducted in accordance with the Institutional Review Board of the School of Management at Harbin Institute Technology.  The IRB approval document does not include an approval number; however, we uploaded the IRB approval document to clarify as a supplementary attachment. |
|  | Informed consent | Describe the informed consent process. Where were the participants told the length of time of the survey, which data were stored and where and for how long, who the investigator was, and the purpose of the study? | The participants were approached by salespersons from three companies, leveraging their own client network, and the online questionnaire was distributed via WeChat. Before answering the survey, participants were informed about the research purpose, research team, ethical clearance information, the anonymity of their responses, and the estimated time to complete the survey. In addition, they were informed that participating in this study has no implications for their relationships with the salespersons or company or employers. If they agree to participate, they can proceed. |
|  | Data protection | If any personal information was collected or stored, describe what mechanisms were used to protect unauthorized access. | The survey was conducted anonymously, the study did not collect any personal privacy or identifying information from participants. The only identifier is a WeChat ID or phone number, which is not related to any personal/employment information. |
| Development and pre-testing |  |  |  |
|  | Development and testing | State how the survey was developed, including whether the usability and technical functionality of the electronic questionnaire had been tested before fielding the questionnaire. | The development of the survey involved the following key steps:  Item Adaptation: The items for the survey were adapted from previous research to ensure validity and contextual relevance. A five-point Likert scale was used for responses, ranging from “strongly disagree” to “strongly agree.”  Translation: To ensure clarity for the target participants, the instrument was translated from English to Chinese using a back-translation method. Three experts in the Information Systems field evaluated the clarity, wording, ease of understanding, and validity of the questionnaire. Minor modifications were recommended to improve item wording and sequence.  Pilot Study: A pilot study involving 40 physicians was conducted to test the usability and clarity of the revised questionnaire.  Additionally, the study uses the Wenjuanxing ([www.wjx.com](http://www.wjx.com)) platform to design the questionnaire.  Wenjuanxing (Questionnaire Star) is an online platform that allows users to design, distribute, and analyze surveys. It provides a user-friendly interface for creating electronic questionnaires, with various customizable options for question types, logic settings, and design layouts. The platform supports both mobile and desktop access, ensuring that respondents can complete surveys on different devices, such as the WeChat platform. Wenjuanxing also offers data collection, analysis tools, and export options, making it convenient for researchers to manage survey data. |
| Recruitment process and description of the sample having access to the questionnaire |  |  |  |
|  | Open survey versus closed survey | An “open survey” is a survey open for each visitor of a site, while a closed survey is only open to a sample which the investigator knows (password-protected survey). | Physicians accessed the survey by scanning a unique QR code or link provided for the questionnaire. Participants could only see the questionnaire he/she has completed himself/herself. |
|  | Contact mode | Indicate whether or not the initial contact with the potential participants was made on the Internet. (Investigators may also send out questionnaires by mail and allow for Web-based data entry.) | The initial contact with potential participants was not made directly on the Internet by the investigators. Instead, a snowball sampling method was used to recruit medication salespersons as intermediaries. Each salesperson assisted by distributing the electronic questionnaire to at least 10 physicians. |
|  | Advertising the survey | How/where was the survey announced or advertised? Some examples are offline media (newspapers), or online (mailing lists – If yes, which ones?) or banner ads (Where were these banner ads posted and what did they look like?). It is important to know the wording of the announcement as it will heavily influence who chooses to participate. Ideally the survey announcement should be published as an appendix. | The survey was not announced or advertised through offline media, online mailing lists, or banner ads. |
| Survey administration |  |  |  |
|  | Web/E-mail | State the type of e-survey (eg, one posted on a Web site, or one sent out through e-mail). If it is an e-mail survey, were the responses entered manually into a database, or was there an automatic method for capturing responses? | It is an e-survey ([www.wjx.com](http://www.wjx.com)), and it has a QR code. People who scan the QR code can complete the survey. The survey consists of multiple-choice questions (Likert scale), and participants select their answers manually. |
|  | Context | Describe the Web site (for mailing list/newsgroup) in which the survey was posted. What is the Web site about, who is visiting it, what are visitors normally looking for? Discuss to what degree the content of the Web site could pre-select the sample or influence the results. For example, a survey about vaccination on a anti-immunization Web site will have different results from a Web survey conducted on a government Web site | The survey was not posted on any specific website, mailing list, or newsgroup. Instead, it was distributed through snowball sampling, where medication salespersons were recruited to share the electronic survey with physicians.  Wenjuanxing (Questionnaire Star) is only a tool for editing electronic questionnaires. Therefore, there is no web-based platform associated with the survey that could influence the sample or results based on its content or typical visitor demographics. |
|  | Mandatory/voluntary | Was it a mandatory survey to be filled in by every visitor who wanted to enter the Web site, or was it a voluntary survey? | Participants were informed that their participation in the study was optional, and there were no requirements for them to complete it in order to access any website or service.  They can voluntarily stop or withdraw from filling out the questionnaire. |
|  | Incentives | Were any incentives offered (eg, monetary, prizes, or non-monetary incentives such as an offer to provide the survey results)? | No incentives were offered to participants. |
|  | Time/Date | In what timeframe were the data collected? | The data were collected over the course of one month. |
|  | Randomization of items or questionnaires | To prevent biases items can be randomized or alternated. | We make randomization of items. |
|  | Adaptive questioning | Use adaptive questioning (certain items, or only conditionally displayed based on responses to other items) to reduce number and complexity of the questions. | Not applicable. All items are supposed to be answered. |
|  | Number of Items | What was the number of questionnaire items per page? The number of items is an important factor for the completion rate. | Respondents can easily navigate through the questions by scrolling on the phone screen. With a total of 39 items and a maximum of 10 items per phone screen. This layout should help maintain engagement and improve completion rates. |
|  | Number of screens (pages) | Over how many pages was the questionnaire distributed? The number of items is an important factor for the completion rate. | Respondents can easily navigate through the questions by scrolling on the phone screen. With a total of 39 items and a maximum of 10 items per phone screen. This layout should help maintain engagement and improve completion rates. |
|  | Completeness check | It is technically possible to do consistency or completeness checks before the questionnaire is submitted. Was this done, and if “yes”, how (usually JAVAScript)? An alternative is to check for completeness after the questionnaire has been submitted (and highlight mandatory items). If this has been done, it should be reported. All items should provide a non-response option such as “not applicable” or “rather not say”, and selection of one response option should be enforced. | In the pilot study, we have ruled out the possibility of having a “not applicable” situation. In the questionnaire, we did not include those options. However, if participants do not want to answer or cannot answer, they can stop or submit the questionnaire at any time. There is no requirement for participants to complete all the questions. At the end of data collection, we manually examined all responses to identify the valid ones for data analysis and hypothesis testing. |
|  | Review step | State whether respondents were able to review and change their answers (eg, through a Back button or a Review step which displays a summary of the responses and asks the respondents if they are correct). | During the survey, participants could easily navigate to answer the questionnaire, but after submission, they were not permitted to review or change their answers. Respondents provided answers based on their personal feelings and experiences, as there were no standardized correct answers for the questions. |
| Response rates |  |  |  |
|  | Unique site visitor | If you provide view rates or participation rates, you need to define how you determined a unique visitor. There are different techniques available, based on IP addresses or cookies or both. | Although we do not collect respondents' personal phone numbers, to distinguish between respondents, we set up the electronic questionnaire distribution so that each phone number can only complete the survey once. Each doctor can only complete the survey using their own mobile phone via WeChat.  This unique identification is automatically completed by the electronic questionnaire website. |
|  | View rate (Ratio of unique survey visitors/unique site visitors) | Requires counting unique visitors to the first page of the survey, divided by the number of unique site visitors (not page views!). It is not unusual to have view rates of less than 0.1 % if the survey is voluntary. | There are no unique site visitors in our paper.  We distributed the questionnaire directly to physicians using a snowball sampling method, screening which physicians had used online health platforms with the first question. It was difficult to determine the total number of physicians to whom we distributed the questionnaire; we could only obtain the number of participants who exited at the first question. 244 physicians exited from the screening question. We collected a total of 327 completed questionnaires.  A total of 327 completed questions were collected within one month. After filtering out invalid responses, such as those with no response variance, incomplete answers, or contradictory responses, 257 valid responses were retained for data analysis and hypothesis testing. The process of collecting our study 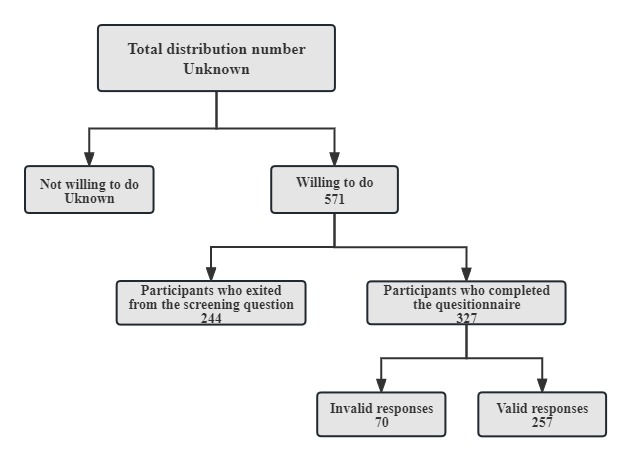sample can be seen in the diagram below. |
|  | Participation rate (Ratio of unique visitors who agreed to participate/unique first survey page visitors) | Count the unique number of people who filled in the first survey page (or agreed to participate, for example by checking a checkbox), divided by visitors who visit the first page of the survey (or the informed consents page, if present). This can also be called “recruitment” rate. |  |
|  | Completion rate (Ratio of users who finished the survey/users who agreed to participate) | The number of people submitting the last questionnaire page, divided by the number of people who agreed to participate (or submitted the first survey page). This is only relevant if there is a separate “informed consent” page or if the survey goes over several pages. This is a measure for attrition. Note that “completion” can involve leaving questionnaire items blank. This is not a measure for how completely questionnaires were filled in. (If you need a measure for this, use the word “completeness rate”.) |  |
| Preventing multiple entries from the same individual |  |  |  |
|  | Cookies used | Indicate whether cookies were used to assign a unique user identifier to each client computer. If so, mention the page on which the cookie was set and read, and how long the cookie was valid. Were duplicate entries avoided by preventing users access to the survey twice; or were duplicate database entries having the same user ID eliminated before analysis? In the latter case, which entries were kept for analysis (eg, the first entry or the most recent)? | We used Wenjuanxing to create the questionnaire, and after completing it, a QR code was generated. We can distribute the QR code to our target audience, who can scan it with WeChat to access the questionnaire. The system will automatically recognize their WeChat ID or phone number as a unique identification marker, allowing each phone number or WeChat ID to enter the questionnaire only once. We cannot access participants' WeChat IDs or phone numbers; we can only obtain the participant identification numbers automatically generated by the system. We can only obtain data from participants who submit the questionnaire.  So, the data we got is unique. |
|  | IP check | Indicate whether the IP address of the client computer was used to identify potential duplicate entries from the same user. If so, mention the period of time for which no two entries from the same IP address were allowed (eg, 24 hours). Were duplicate entries avoided by preventing users with the same IP address access to the survey twice; or were duplicate database entries having the same IP address within a given period of time eliminated before analysis? If the latter, which entries were kept for analysis (eg, the first entry or the most recent)? | We rely on WeChat ID or phone number in this study. IP tracking is not applicable. |
|  | Log file analysis | Indicate whether other techniques to analyze the log file for identification of multiple entries were used. If so, please describe. | This is not applicable. WeChat ID or phone number is used as an identifier to detect the duplication. Moreover, each participant can only participate once. Without economic incentives, the likelihood of using multiple WeChat IDs to participate is slim. |
|  | Registration | In “closed” (non-open) surveys, users need to login first and it is easier to prevent duplicate entries from the same user. Describe how this was done. For example, was the survey never displayed a second time once the user had filled it in, or was the username stored together with the survey results and later eliminated? If the latter, which entries were kept for analysis (eg, the first entry or the most recent)? | As mentioned above, the system will automatically recognize their WeChat ID or phone number as a unique identification marker, allowing each phone number or WeChat ID to complete the questionnaire only once. |
| Analysis |  |  |  |
|  | Handling of incomplete questionnaires | Were only completed questionnaires analyzed? Were questionnaires which terminated early (where, for example, users did not go through all questionnaire pages) also analyzed? | We didn’t include incomplete responses in the analysis because such responses also show no variance in major variables, and/or contradictory responses. Hence, such responses are deemed invalid. The examination of the demographic information did not show any obvious pattern or significant group difference from the valid responses. So, excluding such responses will not affect the results. |
|  | Questionnaires submitted with a typical timestamp | Some investigators may measure the time people needed to fill in a questionnaire and exclude questionnaires that were submitted too soon. Specify the timeframe that was used as a cut-off point, and describe how this point was determined. | To ensure the quality of the data collected, we established a cut-off time of 2 minutes for questionnaire completion. This threshold was determined based on pilot testing, which indicated that the average time to complete the survey was approximately 6 minutes. Questionnaires submitted in less than 2 minutes were excluded from the analysis to eliminate responses that may not reflect thoughtful engagement. |
|  | Statistical correction | Indicate whether any methods such as weighting of items or propensity scores have been used to adjust for the non-representative sample; if so, please describe the methods. | No, we do not use any methods to adjust items. All measures used in this study are reflective rather than formative. |
