# Supplementary material for: Impact of Reverse Empowerment and Proactive Motivations on Physicians’ Online Knowledge Sharing in Digital Platforms: Survey Study
Source: J Med Internet Res. 2024 Nov 29;26:e59904. doi: 10.2196/59904 (PMC11661403; doi:10.2196/59904)
Supplement: Multimedia Appendix 2 [file jmir_v26i1e59904_app2.docx]

**Multimedia Appendix 2**

**Table.** **Measurement Scales**

| Constructions | Items |
| --- | --- |
| Patient empowering behaviors | PEB1 The patients helped me understand the meaning of my contribution to them.  PEB2 The patients helped me see the importance of my contribution to them.  PEB3 The patients made health-related decisions together with me.  PEB4 The patients consulted me on decisions about their health.  PEB5 The patients believed that I could handle demanding tasks.  PEB6 The patients expressed confidence in my contribution.  PEB7 The patients allowed me to contribute knowledge my way. |
| Knowledge sharing self-efficacy | SE1 I have confidence in my ability to provide health knowledge that the patients in the online health platform consider valuable.  SE2 I have the expertise, experience, and insights needed to provide health knowledge that is valuable for the patients in the online health platform.  SE3 I have confidence in responding to messages posted by the patients on the online health platform. |
| Sharing meaning | SM1 My online knowledge sharing is meaningful.  SM2 My online knowledge sharing is very important to me.  SM3 My online knowledge-sharing behaviors are personally meaningful to me. |
| Positive affect | Online knowledge sharing with patients makes me feel my profession …  PA1 Enthusiastic  PA2 Interested  PA3 Determined  PA4 Excited  PA5 Active  PA6 Proud  PA7 Inspired  PA8 Strong  PA9 Alert*  PA10 Attentive |
| Knowledge sharing | In the online health platforms,  KS1 I intend to share knowledge with patients.  KS2 I am always trying my best to share knowledge with patients.  KS3 I am always making an effort to share knowledge with patients.  KS4 I am always willing to share knowledge with patients when they ask. |
| Proactive personality | PERP1 No matter what the odds are, if I believe in something, I will make it happen.  PERP2 I love being a champion for my idea, even against others’ opposition.  PERP3 I am excellent at identifying opportunities. *  PERP4 If I believe in an ideal, no obstacle will prevent me from making it happen. |
| Extrinsic rewards | ER1 Sharing knowledge can enhance my reputation in the online health platform.  ER2 I get praise from others by sharing knowledge on the online health platform.  ER3 I feel that knowledge sharing improves my status in the online health platform. |
| *Deleted item. | |
